# Jointly learning word embeddings using a corpus and a knowledge base

**DOI:** 10.1371/journal.pone.0193094

**Published:** 2018-03-12

**Authors:** Mohammed Alsuhaibani, Danushka Bollegala, Takanori Maehara, Ken-ichi Kawarabayashi

**Affiliations:** 1 Department of Computer Science, University of Liverpool, Liverpool, United Kingdom; 2 RIKEN Center for Advanced Intelligence Project, Tokyo, Japan; 3 National Institute of Informatics, Tokyo, Japan; 4 Kawarabayashi ERATO Large Graph Project, Tokyo, Japan; University of Lisbon, PORTUGAL

## Abstract

Methods for representing the meaning of words in vector spaces purely using the information distributed in text corpora have proved to be very valuable in various text mining and natural language processing (NLP) tasks. However, these methods still disregard the valuable semantic relational structure between words in co-occurring contexts. These beneficial semantic relational structures are contained in manually-created knowledge bases (KBs) such as ontologies and semantic lexicons, where the meanings of words are represented by defining the various relationships that exist among those words. We combine the knowledge in both a corpus and a KB to learn better word embeddings. Specifically, we propose a *joint* word representation learning method that uses the knowledge in the KBs, and simultaneously predicts the co-occurrences of two words in a corpus context. In particular, we use the corpus to define our objective function subject to the relational constrains derived from the KB. We further utilise the corpus co-occurrence statistics to propose two novel approaches, Nearest Neighbour Expansion (NNE) and Hedged Nearest Neighbour Expansion (HNE), that dynamically expand the KB and therefore derive more constraints that guide the optimisation process. Our experimental results over a wide-range of benchmark tasks demonstrate that the proposed method statistically significantly improves the accuracy of the word embeddings learnt. It outperforms a corpus-only baseline and reports an improvement of a number of previously proposed methods that incorporate corpora and KBs in both semantic similarity prediction and word analogy detection tasks.

## Introduction

Understanding the meanings of words is an essential step for natural language processing (NLP) systems. In recent years, there has been an immense interest in methods that learn word (meaning) representations in an unsupervised manner from massive text collections. Such methods often represent the meanings of words in linear algebraic structures such as vectors that capture lexico-semantic information about the word. The usefulness of such word embeddings has been demonstrated by their impressive performances in various NLP tasks, such as name entity recognition (NER) [1], word similarity measurement [2], sentiment analysis [3], word analogy detection [4], syntactic parsing [5] and dependency parsing [6]. Moreover, high-quality embeddings of individual words can be used to build semantic representations for larger lexical units such as phrases, sentences and documents in a bottom-up manner by recursively applying semantic compositional operators on the word-level embeddings [7].

Two main approaches can be identified in prior work on word embeddings learning: (a) Corpus-based and (b) KB-based approaches. Despite the many success stories of data-driven corpus-based approaches for learning word embeddings in various NLP tasks, those approaches operate on surface-level word co-occurrences, ignoring the rich semantic relations between two words encoded in KBs such as semantic lexicons. KBs-based approaches provide an alternative solution for representing the meanings of words considering the relations such as synonymy, hypernymy and meronymy that exist between words. For example, in the WordNet [8], the word *dog* has a hypernymic relation with its superclass *pets*. Such information in the KBs are an invaluable source for learning better word embeddings when it is blended with corpus-based approaches [9, 10]. For example, the corpus-based approaches rely on the occurrences of words in the corpus which can be ambiguous, whereas KBs typically group words that have similar senses (eg. WordNet synsets). Moreover, it can be problematic when learning word embeddings purely from a corpus when those words are rare, because the corpus might not be sufficiently large to obtain reliable co-occurrences counts.

Although KBs provide valuable information about words relations, such information is manually curated and thus costly to produce. Therefore, learning word embeddings purely from KBs, without considering the rich contextual information that exist in text corpora has several limitations. For example, in a KB, a particular word often has a limited number of entries, which makes it difficult to estimate the strength of the relation between two words. However, in a corpus, we can observe numerous co-occurrences between two words in different contexts. The absence of contextual information of a word in a KB is a disadvantage when applying distributional approaches for learning word embeddings. Moreover, new words or novel uses of existing words (eg. neologisms and semantic extensions) are not very well covered by the manually constructed and maintained KBs. In contrast, a text corpus is likely to capture such dynamic and temporal linguistic phenomena.

Considering the above-mentioned complementary strengths when learning word embeddings using the two types of resources, text corpora and KBs, the following question naturally arises: *can we learn higher-quality word embeddings by using text corpora and KBs simultaneously than using only one of those resources*?

As a concrete example of how a KB can complement a corpus to learn better word embeddings, let us assume that the words, *dog* and *cat* are recorded in the KB as instances of the word *pet*, possibly via an IS-A relation. Further assume that the sentence “*I like both cats and dogs*” is the only sentence in the corpus where the three words *cat*, *dog* and *like* occur. Therefore, if we were to use a corpus-only approach, we would learn word embeddings for those three words that predict the similarity between *cat* and *dog* to be equal to that between *cat* and *like*, because there is only one sentence containing all three target words. However, in a KB we might find that *cat* and *dog* are listed as hyponyms of *pet*, but not of *like*. Therefore, such constraints provided by the KB can potentially solve the sparse co-occurrence problem encountered in corpus-only approaches for learning word embeddings.

Our main contribution in this paper is a word embedding learning method that uses both a corpus and a KB in a *joint* manner. The proposed method instigates by randomly initialising the word embeddings with real-valued, fixed, low-dimensional vectors, which are subsequently updated such that the co-occurrences between words in a corpus can be accurately predicted using the learnt embeddings. For this purpose, we extend the objective function of the Global Vectors (GloVe) [11] by incorporating the knowledge in the KB as a constraint in the optimisation. Specifically, if two words have a particular semantic relationship in the KB, then we require their word embeddings to be similar.

In practice, the KB might be incomplete and inconsistent with the information available in the corpus with which we combine for learning word embeddings. To overcome such disfluencies in the KB, we extend our prior work [9] by proposing several strategies to *dynamically* update the KB with information extracted from the corpus to learn better word embeddings via a joint approach. Specifically, we consider two approaches for expanding the KB by considering the nearest neighbours of a word in the corpus, which we referred to as the Nearest Neighbour Expansion (NNE) and Hedged Nearest Neighbour Expansion (HNE). Our experimental results show that in comparison to not expanding the KB (which we referred to as the Static Knowledge Base (SKB)) both NNE and HNE methods help us learn higher-quality word embeddings. Interestingly, by iterating the expansion process, we show that the accuracy of the learnt word embeddings can be further improved.

In our experiments, we use eight different relation types extracted from WordNet. Experimental results on two standard NLP tasks, semantic similarity prediction [12] and word analogy prediction [13] show substantial improvements on the accuracy of the word embeddings learnt by the proposed method. On both tasks, our proposed method using SKB, NNE and HNE statistically significantly outperform the corpus-based baseline and reports an improvement of a number of previously proposed methods that incorporate corpora and KBs for learning word embeddings. We empirically study the effect of the dimensionality of the embeddings, size of the corpus and the KB on the accuracy of the word embeddings learnt. The proposed KB expansion methods can be applied repeatedly with the learnt word embeddings to find better expansion candidates for the KB. Interestingly, we see that by repeatedly applying the proposed method we can further improve the accuracy of the learnt word embeddings.

The proposed joint model can be utilised to be applied to various domains. For instance, a line of research has recently shown that learning accurate word or term representations is an important task in the biomedical domain. For example, [14–16] show that it is possible to learn cross-lingual word embeddings from UMLS [17] Metathesaurus to find translations for biomedical terms. In biomedical domain, there are large scale unstructured corpora such as Medline (https://www.nlm.nih.gov/databases/) corpus, which have been extensively used for text mining tasks. On the other hand, rich ontologies such as Snomed-CT (http://www.snomed.org/snomed-ct/) are also available for representing meanings of technical terms. An interesting future research direction would be to use our proposed method to utilise both ontologies and corpora available in the biomedical domain to learn better word/term representations. Moreover, the view of combining KBs/ontologies and corpora in a joint learning fashion has been recently adopted in the biomedical domain to extract biomedical relation. For example, [18] proposed a distant supervision approach that utilise various text corpora and ontologies for the task of microRNA-gene relations extraction from biomedical literature. As such, using our proposed method to learn word/term representations to assist such tasks will be another interesting future direction.

## Related work

Unsupervised approaches for learning word embeddings from large text corpora have received much attention lately. SOTA performances in a variety of NLP tasks have been reported by using word embeddings as features [1, 19]. Continuous bag-of-words model (CBOW) and skip-gram model (SG) [20] are two popular word embedding learning methods that leverage the local co-occurrences between words in a corpus. Given context words in some co-occurrence context window, CBOW predicts a target word, whereas SG, per contra, predicts the context words given the target word. In contrast, GloVe [11] first builds a global word-word co-occurrence matrix, and then predicts the global co-occurrence count between two words (target and context) using the corresponding word embeddings. Despite their success, the above-mentioned methods use only a corpus as the sole data source to learn word embeddings.

On the other hand, Relation Constrained Model (RCM) [21] incorporates knowledge from a KB in the form of word similarity scores, into the CBOW word embedding learning objective, where similar words in the KB are assigned with high sampling probabilities. Specifically, their proposed method instigate by employing the CBOW objective function with the replacement of the context information by relational data found in the KB. Next, throughout a linear combination between the two objectives (CBOW and RCM) they form a joint model utilizing the two sources of data. Similarly, RC-NET [22] jointly learns word embeddings using the SG objective combined with a KB. RC-NET considers both relational (R-NET) and categorical (C-NET) information and represents both words and relations in the same embedding space. Particularly, R-NET incorporates the relational knowledge throughout a regularization function that considers the relationships between entities as translations on the low-dimensional representations of the entities. For C-NET, another regularization function is defined to leverage the categorical information by minimising the weighted distance between shared-attributes words. Next, SG, R-NET and C-NET objectives are combined and trained with backpropagation to learn the word embeddings. Liu et al. [23] proposed a method that represents the semantic knowledge in KBs as word ordinal ranking inequalities, which are subsequently used as constraints in the SG objective.

Although we share a similar motivation to the above-mentioned joint approaches for learning word embeddings, our proposed method differs from those prior proposals in several aspects. Firstly, CBOW and SG are the base training objectives for the above models whereas we adopt GloVe as our corpus-based objective. As such, no costly normalisation over the entire vocabulary or negative sampling are required with the proposed method. Specifically, from a computational point of view, CBOW requires normalizing the output probabilities of target words over the entire vocabulary, which is often very large. Consequently, approximate methods such as hierarchical softmax has been proposed to overcome this problem [24]. On the other hand, GloVe does not require such expensive normalizations. Furthermore, in contrast to the aforementioned joint models which consider only the original data existed in the KBs, our proposed method further enhances the joint process by dynamically expand the KB using the corpus co-occurrence statistics.

A complementary research direction to us focuses on incorporating the information in KBs into the pre-trained word embeddings, trained purely from a corpus, in a post-processing step. For example, *retrofit* is an efficient post-processing step that can fit pre-trained embeddings from any word embedding learning method to a semantic lexicon that lists pairs of words belonging to a particular semantic relation [10]. Johansson and Piña [25] proposed a method to obtain word sense embeddings by fitting pre-trained word embeddings to a semantic network. Although the post-processing models have various advantages such as that they can employ any corpus-based word embeddings to fit to a KB, such post-processing approaches do not jointly leverage the KB during the word representations learning phase because only the corpus is used for learning the pre-trained word embeddings.

Goikoetxea et al. [26] proposed a method that performs a truncated random walk in the KB graph to generate a pseudo corpus by sequentially recording the words that were visited during the random walk. Next, corpus-based word embedding learning methods are used to learn representations from this pseudo corpus. Unfortunately, this approach is limited to learning word representations only from a KB, ignoring any text corpora that mights co-exist alongside the KB. Bollegala et al. [4] use a relational graph to learn word embeddings. They represent words by vectors and relations by matrices. Their method can operate on either a manually created or automatically extracted relational graphs. However, their KB remains fixed throughout the training process, and they do not update the KB with the information found in the corpus as we propose in this paper. As shown later in our experiments, by dynamically updating the KB with the information from the corpus we can learn better word representations.

Another body of work on jointly utilizing both unstructured text corpora and manually created KBs focus on learning relation and knowledge representations. Toutanova et al. [27] proposed a method that combines a given KB that lists the relations between entities and lexical relations extracted from a corpus using a dependency parsing and then jointly learn continuous representation of KB, lexical relations and entities for the tasks of link prediction and relational extraction. Wang and Li [28] proposed a model that learns a knowledge graph representation by leveraging contextual information in a corpus. They instigate by annotating the entities in the corpus, then construct a co-occurrence network between words and entities and finally an optimization procedure is employed to learn embeddings for entities and relations.

## Learning word embeddings

We propose a method to learn word embeddings from both a corpus and a KB in a *joint* manner. First, in the “Global Vectors (GloVe)” section, we briefly review GloVe, which forms the basis of the corpus-based objective in our proposed method. Next, in the “Incorporating the Knowledge Base” section, we describe the derivation of constraints from a KB. Finally, in the “Joint Objective Function” section, we detail the joint learning method.

### Global Vectors (GloVe)

GloVe [11] learns continuous word vectors from a text corpus by leveraging statistical information computed from a global word co-occurrence matrix. In particular, given a corpus C, GloVe instigates by creating a co-occurrence matrix **X**, where each *target word* (i.e. the word that we want to learn a representations for) is represented by a row in **X**, and the *context words* that co-occur with it in some contextual window, are represented by the columns of **X**. The entries *X*_*ij*_ denote the total occurrences of target word *w*_*i*_ and the context of word ${\tilde{w}}_{j}$ in the corpus. Next, for each word *w*_*i*_ in the vocabulary V (i.e., the set of all words in the corpus), GloVe seeks to learn word embedding $w_{i},{\tilde{w}}_{i}\in R^{d}$ corresponding respectively to whether *w*_*i*_ is a target word or a context word ${\tilde{w}}_{i}$. The boldface ***w***_*i*_ denotes the word embedding (vector) of the word *w*_*i*_, and the dimensionality *d* is a user-specified hyperparameter. The GloVe embedding learning method minimises the following weighted least squares loss:

$$\begin{array}{c} J_{C}=\frac{1}{2}\sum_{i\in V}\sum_{j\in V}f\left(X_{ij}\right){\left({w_{i}}^{⊤}{\tilde{w}}_{j}+b_{i}+{\tilde{b}}_{j}-\text{log}\left(X_{ij}\right)\right)}^{2} \end{array}$$ (1)

Here, the two real-valued scalars *b*_*i*_ and ${\tilde{b}}_{j}$ are biases associated respectively with *w*_*i*_ and ${\tilde{w}}_{j}$. The weighting function *f* assigns a lower weight for extremely frequent co-occurrences to prevent over-emphasising such co-occurrences, and is given by:

$$\begin{array}{c} f\left(t\right)=\{\begin{array}{cc} {\left(t/t_{\max}\right)}^{\alpha } & \text{if}\;t<t_{\max}\\ 1 & \text{otherwise} \end{array} \end{array}$$ (2)

The GloVe objective function defined by (1) attempts to predict the co-occurrence between two words *w*_*i*_ and ${\tilde{w}}_{j}$ using the inner-product between the corresponding vectors ***w***_*i*_ and ${\tilde{w}}_{j}$. Those vectors are learnt such that the squared difference between the inner-product and the logarithm of their co-occurrence count is minimised. Mikolov et al. [29] showed that the vector equation *king*—*queen* = *man*—*woman* approximately holds, for the embeddings of the four words *king*, *queen*, *man* and *women*. This empirical result implies that we can use the difference between two word embeddings as a proxy for the representation for the relationship between those words. Eq 1 is constructed such that the learnt words embeddings represent the relationship between two words by their vector difference (offset) [30].

### Incorporating the knowledge base

GloVe is a corpus-only word embedding method that does not leverage any available KBs. Therefore, it is likely to encounter problems when learning word embeddings from rare co-occurrences and may fail to capture the desired semantics. To address this problem, we derive constraints from the KB that must be satisfied by the learnt word embeddings. Given a KB S, we define an objective $J_{S}$ that considers not only two-way co-occurrences between a target word *w*_*i*_ and one of its context words ${\tilde{w}}_{j}$ but rather a three-way co-occurrence between *w*_*i*_, ${\tilde{w}}_{j}$ and the semantic relations *R* that exists between them in the KB. Although we use WordNet as a concrete example of a KB in this work, there are no assumptions made regarding any structural properties unique to a particular KB. Any KB that defines semantic relations between words can be used as S, such as FrameNet [31] and the Paraphrase Database (PPDB) [32] can be used with the proposed method. The KB-based objective is defined as follows:

$$\begin{array}{c} J_{S}=\frac{1}{2}\sum_{i\in V}\sum_{j\in V}R\left(w_{i},w_{j}\right){\left(w_{i}-{\tilde{w}}_{j}\right)}^{2} \end{array}$$ (3)

Here, $R(w_{i},{\tilde{w}}_{j})$ indicates the strength of the relation *R* between *w*_*i*_ and ${\tilde{w}}_{j}$. If *R* does not hold between the two words, then $R(w_{i},{\tilde{w}}_{j})$ is set to zero. Note that in a typical KB we might encounter a large number of different relation types. Eq 3 is not limited to a particular relation type or number of relation types and can easily be extended to handle multiple relation types *R*_*r*_ as follows:

$$\begin{array}{c} J_{S}=\frac{1}{2}\sum_{r\in R}\sum_{i\in V}\sum_{j\in V}R_{r}(w_{i},w_{j}){\left(w_{i}-{\tilde{w}}_{j}\right)}^{2} \end{array}$$ (4)

For the simplicity of the disposition, we would limit our discussion here to KBs where there exist only one type of semantic relation between two words. In our experiments we show results on a wide-range of different relation types. Without loss of generality, the semantic relations are assumed to be asymmetric. In other words, we have $R\left(w_{i},{\tilde{w}}_{j}\right)\neq R\left({\tilde{w}}_{j},w_{i}\right)$. Both types of relations, symmetric (e.g. synonymy and antonymy) and asymmetric (e.g. hypernymy and meronymy) are considered in our experiments. Eq 3 formalises the constraint that the words which are connected in the KB by some semantic relations *R* must have similar word representations.

### Joint objective function

To simultaneously minimise both the Eqs 1 and 3, we defined a combined objective as their linearly weighted combination given by,

$$\begin{array}{c} J=J_{C}+\lambda J_{S}. \end{array}$$ (5)

Here, λ ∈ ℝ^+^ is a regularisation coefficient that controls the influence imparted by the KB on the word embeddings learnt from the corpus. Details of estimating the optimal value of λ is described later in the “Evaluation” section.

The overall joint objective function given by Eq 5 is convex w.r.t. each one of the variables ***w***_*i*_, ${\tilde{w}}_{j}$, *b*_*i*_ and ${\tilde{b}}_{j}$, if the other three variables are held fixed. We use an alternating optimisation technique where all the parameters are first randomly initialised and then, in a pre-specified order, cycle through the variables updating one at a time, while the other variables are held fixed.

**Algorithm 1** Joint word embedding learning.

**Input**: Word co-occurrence matrix **X** specifying the co-occurrences between words in the corpus C, relation strength $R(w_{i},{\tilde{w}}_{j})$ specifying the semantic relations between words in the KB S, dimensionality *d* of the word embeddings, and the maximum number of iterations *T*.

**Output**: Embeddings $w_{i},{\tilde{w}}_{j}\in R^{d}$, of all words $w_{i},w_{j}\in V$.

1: Initialize word vectors $w_{i},{\tilde{w}}_{j}\in R^{d}$ randomly.

2: **for** *t* = 1 **to** *T* **do**

3:  **for** (*i*, *j*) ∈ **X** **do**

4:   Use (8) to update ***w***_*i*_

5:   Use (9) to update *b*_*i*_

6:   Use (10) to update ${\tilde{w}}_{j}$

7:   Use (11) to update ${\tilde{b}}_{j}$

8:  **end for**

9: **end for**

10: **return** $w_{i},{\tilde{w}}_{j}\;\;\;\forall w_{i},w_{j}\in V$.

## Dynamic KB expansion

In practice, a KB might not contain all the words in the corpus. Because we derive constraints only from the KB, the coverage of the constraints derived from the KB might cover only a small fraction of the words in the corpus. To overcome this problem, we propose two methods to expand the KB using the information extracted from the corpus. It is noteworthy that the purpose of performing this expansion is to derive constraints that guide the optimisation process and not to build better KBs. Because the expansion of the KB happens at run time, we call it *dynamic* expansion.

### Static Knowledge Base (SKB)

The SKB approach does *not* dynamically expand the KB, and acts as a baseline for comparing against the two dynamic expansion methods we describe in the following sections. Let us assume a KB where knowledge is represented in the form of relational tuples (*u*, *R*, *v*), involving a relation *R* that exists between two words *u* and *v*, each tuple contributes to a single constraint towards the joint objective given by Eq 5. In what follows, we denote the set of vertices (vocabulary) in the KB by D, and its set of relational tuples by E. If two words u,v∈D have a relation *R*, then we have (u,R,v)∈E. For example, for the synonymy relation in the WordNet, we obtained 87,06 tuples. In **SKB**, we assume the relation strength function *R*(*w*_*i*_, *w*_*j*_) given by (3) to be a binary function that returns 1 if there exists a semantic relation *R* between the two words *w*_*i*_ and *w*_*j*_ in the KB S and 0 otherwise.

### Nearest Neighbour Expansion (NNE)

Typically, a corpus would cover a much larger vocabulary and more relations can be derived from it as compared to that by a KB. If we can somehow use the information extracted from the corpus to dynamically expand the KB, then we can derive more constrains for the joint optimisation process, thereby making a better use of the KB. If two words *u* and *v* co-occur frequently in a corpus, then it is likely that there exists some semantic relation between those two words. We can compute the strength of association between two words using their co-occurrence count in the corpus, to create a k-nearest neighbour (K-NN) graph where the *u* is connected to *v* if and only if *v* is among the top-k nearest neighbours of *u*. In our experiments, we use the Positive Pointwise Mutual Information (PPMI) [33] as the association measure, and selected the top-K neighbours according to the highest PPMI values between two words. Denoting the co-occurrence count between *u* and *v* in the corpus by *c*(*u*, *v*) and the occurrence of *u* and *v* respectively by *c*(*u*, *) and *c*(*, *v*), the PPMI between *u* and *v*, *PPMI*(*u*, *v*) is computed as follows:

$$\begin{array}{c} \text{PPMI}\left(u,v\right)=\max(\text{log}(\frac{c(u,v)c(*,*)}{c(u,*)c(*,v)}),0) \end{array}$$ (6)

Let us denote the set of *k* nearest neighbours of *u* in the corpus by *K*_*NN*_(*u*). Between a word *u* that occurs in the KB and a word *v* that only occurs in the corpus, if *v* is a nearest neighbour of *u* (i.e. *v* ∈ *k*_*NN*_(*u*)), then we add *v* to the KB. Moreover, the relation between *v* and *u* is set to the default semantic relation of the KB (assuming that the KB is representing one semantic relation type). The relational strength for the appended nearest neighbours are set to their PPMI values with the target word, computed using the corpus co-occurrence counts.

Considering that the nearest neighbours are found from the corpus purely based on co-occurrence statistics, they might not actually be reflecting the same semantic relation as in the KB. Moreover, PPMI values computed from sparse co-occurrences can be unreliable. In contrast, the KB might be a cleaner and an accurate semantic resource that is manually created and maintained. Therefore, we must impose a higher level of confidence on the original words and relations described in the KB than the candidates we automatically append from the corpus. To prioritise the words that originally appeared in the KB over the automatically added words from the corpus, we set the relational strength *R*(*u*, *w*) for two words *u* and *w* that appeared in the KB prior to dynamic expansion to 1. Meanwhile, the relational strength *R*(*u*, *v*) for a word *u* that originally appeared in the KB and an expansion candidate *v* selected from the corpus is set to the normalised PPMI value between *u* and *v*, where we normalise the PPMI values by the sum of PPMI values over all *k* nearest neighbours. Specifically, the relational strength *R*(*u*, *v*) for two words in the KB after the dynamic expansion process is defined as follows:

$$\begin{array}{c} R\left(u,v\right)=\{\begin{array}{cc} 1, & \text{if}\;(u,R,v)\in E\\ \frac{\text{PPMI}(u,v)}{\sum_{v^{\prime }\in K_{NN}\left(u\right)}\text{PPMI}\left(u,v^{\prime }\right)}, & u\in D\wedge v\in K_{NN}\left(u\right) \end{array} \end{array}$$ (7)

We call this dynamic expansion method as the Nearest Neighbour Expansion (NNE), and show its pseudo code in Algorithm 2.

**Algorithm 2** Nearest Neighbour Expansion (NNE).

**Input**: Word co-occurrence matrix *X* specifying the co-occurrences between words in the corpus C, a KB S=(D,E) with a vocabulary D and a set of relational tuples E, hyperparameter K specifying the number of nearest neighbours (NN) to consider.

**Output**: S=(D,E)

1: **for** v∈C **do**

2:  **if** ∃u∈D s.t. $v\notin D\wedge v\in K_{NN}\left(u\right)$ **then**

3:   E←E∪{(u,R,v)}

4:   D←D∪{v}

5:  **end if**

6: **end for**

7: **return** S.

### Hedged Nearest Neighbour Expansion (HNE)

One drawback of the NNE method described in previous section is that it considers the neighbourhood *K*_*NN*_(*u*) of each word *u* currently in the KB separately when deciding whether a new word *v* should be linked to *u*. This operation can be problematic due to two reasons. First, some hub words that are associated with more than one word such as *everything* are not suitable as expansion candidates because they lack specificity. PPMI does not necessarily overcome the hub words problem [34]. Second, some words can be ambiguous and if we expand each word individually as done by NNE, we might incorrectly link different senses of a word from the corpus. For example, let us assume that *Apple* and *Microsoft* are connected via COMPETITOR relation in a KB. Moreover, let us assume that *banana* co-occurs highly with *Apple* in the corpus. Because we do not assume the corpus to be sense annotated, we might incorrectly link *Banana* to *Apple* because it is a nearest neighbour of the fruit sense of *Apple* in the corpus.

We propose two modifications to the NNE method to overcome the above-mentioned disfluencies. First, we require a word *v* to be a nearest neighbour of two words *u* and *h* that are already in the KB before we consider *v* to be an expansion candidate for the KB. This requirement will reduce the attachment of noisy co-occurrences. Second, we require some semantic relations to exist between *u* and *h* in the KB before we consider *v* to be an expansion candidate for the KB. In our previous example, *Banana* (*h*) is unlikely to co-occur a lot with *Microsoft* (*u*) in the corpus, therefore *Banana* will not be considered as an expansion candidate. Because of stricter neighbourhood requirement of this method that limits the extent of the expansion, we call it the Hedged Nearest Neighbour Expansion (HNE) method. Once we have identified the expansion candidates satisfying both of those requirements, we will compute the relation strength using Eq 7 and link *v* to both *u* and *h*. The pseudo code for HNE is shown in Algorithm 3. As shown in our experiments, the dynamic expansion methods can be run multiple times to further expand the KBs.

**Algorithm 3** Hedged Nearest Neighbour Expansion (HNE).

**Input**: Word co-occurrence matrix *X* specifying the co-occurrences between words in the corpus C, a KB S=(D,E) with a vocabulary D and a set of relational tuples E, hyperparameter K specifying the number of nearest neighbours (NN) to consider.

**Output**: D (Expanded S)

1: S=(D,E)

2: **for** v∈C **do**

3:  **if** ∃u,h∈D,v∉D s.t. $v\in K_{NN}\left(u\right)\wedge v\in K_{NN}\left(h\right)\wedge \left(u,R,h\right)\in E$ **then**

4:   E←E∪{(u,R,v),(h,R,v)}

5:   D←D∪{v}

6:  **end if**

7: **end for**

8: **return** S.

## Evaluation

### Data pre-processing

We used ukWaC [35], a large English web corpus comprising of ca. 2 billion tokens crawled from the web from .uk domain, as the corpus in our experiments. To investigate the effect of the corpus size on the proposed method, we randomly select sub-corpora of varying sizes as shown in Table 1.

**Table 1:** Sub-corpora selected from ukWaC.

% of ukWaC	Number of tokens	Size
100	2B	XL
70	1.4B	L
40	800M	M
20	400M	S
10	200M	XS

We use WordNet [8] as the KB in our experiments and consider eight different relation types. For synonyms, we generate all the pairwise combinations of words in a given synset to create synonymous word pairs. The list of synonymous word pairs is considered as the synonyms in Table 2. For other relations, we consider two words *u* and *v* connected by a semantic relation *R* if *R* exists between the two synsets encompassing *u* and *v*. Table 2 shows the number of tuples extracted for relation type (SKB) and the size of the KB after expanding with the corpus using NNE and HNE methods. Because of the extra requirements imposed by HNE over NNE, HNE is expected to assign fewer number of expansion candidates than NNE.

**Table 2:** KB size (in no. of edges) for different relation type under different expansion methods with *K* = 5 expansion words.

Relation Type	Edges		
	SKB	NNE	HNE
Synonyms	87,060	108,510	104,123
Antonyms	4,064	7,004	5,325
Hypernyms	119,029	144,199	138,922
Hyponyms	122,926	141,961	138,010
Member-holonyms	11,506	13,716	12,033
Member-meronyms	11,431	12,706	11,651
Part-holonyms	13,082	18,222	16,557
Part-meronyms	13,251	18,191	16,186

### Implementation details

We create a word co-occurrence matrix **X** considering the words that occur at least 20 times in the corpus. Following prior recommendations [36], we set the context window to the 10 tokens preceding and succeeding a target word in a sentence and extract unigrams as context words. Co-occurrences are weighted by the inverse of the distance between the target word and a context word, measured by the number of tokens appearing in between. We adopt a decreasing weighting function using the reciprocal $\frac{1}{d}$ of the distance between two co-occurrences. For example, a context word co-occurring 5 tokens from a target word would contribute to a co-occurrence count of $\frac{1}{5}$. The weighting function given by Eq 2 is computed with *α* = 0.75 and *t*_max_ = 100.

We use stochastic gradient descent (SGD) with learning rate scheduled using AdaGrad [37] as the optimisation method. The overall algorithm of the proposed joint word embedding learning method is listed in Algorithm 1. The word embeddings are randomly initialised to the uniform distribution in the range [−1, +1] for each dimension separately. Experimentally, *T* = 20 iterations was found to be sufficient for the proposed method to converge to a solution. The initial learning rate in AdaGrad is set to 0.01 in all of the experiments.

Algorithm 1 in Line 3 iterates over the nonzero elements in **X**. The estimated overall time complexity for *n* nonzero elements is O(|V|dTn), where |V| denotes the number of words in the vocabulary. Typically, the global co-occurrence matrix is highly sparse, containing less than 0.03% of non-zero entries. It takes around 50 mins. to learn 300 dimensional word representations for |V| = 434,826 words (*n* = 58,494,880) from the ukWaC corpus on a Xeon 2.9GHz 32 core 512GB RAM machine. Note that building the co-occurrence matrix and expanding the KB can be done in a single traversal over the corpus. Specifically, we can maintain a priority queue to select the K-nearest neighbours based on the co-occurrence counts while building the co-occurrence matrix. Therefore, the computational overhead due to dynamic expansion is insignificant in practice. The source code for the proposed method and the embeddings trained using the proposed method are made publicly available at https://github.com/suhaibani/JointReps.

The proposed method learns two embeddings ***w***_*i*_ and ${\tilde{w}}_{i}$ for each word *w*_*i*_, indicating respectively a target and a context embedding. Prior work in learning word embeddings [36] show that the embedding of a word *w*_*i*_ can be better approximated by adding the two embeddings $w_{i}+{\tilde{w}}_{i}$. This additive operation has been also motivated as an ensemble method in [11]. In our experiments, we followed these prior recommendations and create the final embedding for a word by adding its target and context embeddings. In the remaining sections we consider those word embeddings.

### Qualitative analysis

To qualitatively understand the differences among the proposed KB expansion methods, in Table 3 we show randomly selected examples from SKB, NNE and HNE after a single round of expansions. We can see from Table 3 that HNE was able to successfully eliminate some potential noisy expansion words. For example, the words *everything*, *american*, *soon* and *amity* have been associated as expansion words with *autopilot*, *china* and *pineapple* respectively using NNE, but excluded by HNE. Moreover, because we limit the expansion candidates to the top-K neighbours (K = 5), we can see in Table 3 that some words are included in HNE but not in NNE. In such cases, the top five neighbours according to NNE do not meet the HNE requirements.

**Table 3:** Examples of KB expansion using NNE and HNE on synonym relation type. The SKB column denotes the associated synonym words found in WordNet.

Word	Associated Words		
	SKB	NNE	HNE
autopilot	autopilots	everything land copilot assist american	copilot assist software
imagination	imagery resource resourcefulness imaging imaginativeness vision	art originality mind unfettered fascinate	sight picture sense
pineapple	ananas	soon red pineapples pecan mango	fruit pineapples flowers
magyar	hungarian	culture group central re english	romania language culture
china	cathay taiwan chinaware prc	amity europe beijing south shanghai	beijing shanghai bhutan
sulfur	sulphur	fire test oxide hydrogen reference	oxide odor oxygen

### Benchmarks

We evaluate the quality of the word embeddings produced by the proposed method on two standard tasks: word similarity prediction and word analogy detection.

#### Word similarity

In this task, we measure the cosine similarity between word embeddings learnt by a particular method for two words in a benchmark dataset, and compare that against the average similarity ratings given by a group of human annotators for those two words. If there is a high degree of correlation between human similarity ratings and the similarity scores computed using the learnt word embeddings, then we can conclude that the word embeddings capture word semantics as perceived by humans. We use the Spearman’s rank correlation coefficient as the evaluation measure for the word similarity prediction task, and use Fisher transformation to test for statistical significance. We use multiple word similarity benchmark datasets: WordSim353 (**WS**, 353 word-pairs) [38], Rubenstein-Goodenough (**RG**, 65 word-pairs) [39], Miller-Charles (**MC**, 30 word-pairs) [40], rare words dataset (**RW**, 2034 word-pairs) [41], Stanford’s contextual word similarities (**SCWS**, 2023 word-pairs) [42], **MEN** test collection (3000 word-pairs) [43] and the SimLex-999 (**SimLex**, 999 word-pairs) [44].

#### Word analogy

The vector difference (offset) between embeddings for two words has shown to represent the relationship between those words [24]. Consequently, prior work on word embedding learning has evaluated the accuracy of the trained word embeddings by using them to solve word analogy problems. For this purpose, we use two benchmark datasets: Google dataset [24], and SemEval 2012 Task 2 dataset [45] (**SemEval**). Google dataset contains five semantic (**sem**) and nine syntactic (**sen**) analogy types where (**sem**) consists of 10,675 questions and (**syn**) consists of 8869 questions. SemEval dataset contains 3218 manually ranked word-pairs for 79 paradigms (categories). Given a proportional analogy *a*:*b*::*c*:*d*, we compute the cosine similarity between the ***b*** − ***a*** + ***c*** and each candidate word ***d***, and select the most similar candidate word as the answer to the analogy question. We use binomial exact test with Clopper-Pearson confidence interval to test for the statistical significance. For SemEval, we report the MaxDiff scores using the official evaluation tool (https://sites.google.com/site/semeval2012task2/).

#### Validation set

We use the **WS** dataset as validation data for tuning λ in Eq 5 and the neighbourhood size *K* in Algorithms 2 and 3. Specifically, we vary the value of λ and *K*, use the proposed method for learning word embeddings, and measure the Spearman correlation on **WS**. Finally, we select the hyperparameter values that maximises the Spearman correlation. Overall, we observed that λ = 10,000 and *K* = 5 found to perform consistently well for all semantic relation types. The process was done for all word similarity and analogy benchmarks.

## Results

### Outline

In the “Effectiveness of Joint Learning” section, we first evaluate the benefit of using both a corpus and a KB jointly for learning word embeddings covering a wide range of relation types. Next, in the “Effectiveness of Dynamic Expansion” section, we evaluate the benefit of dynamic expansion. We investigate the effect of the corpus and KB size on the proposed method respectively in the “Effect of the Corpus Size” and “Effect of the KB size” sections. Furthermore, we observe the impact of multi-rounds of expansions using NNE and HNE in the “Multi-Rounds of Expansion” section. Finally, in the “Effect of Dimensionality” section, we report the impact of the dimensionality *d* on the word embeddings learnt. The best performance for each task in each of the upcoming results tables is shown in bold and statistical significance is indicated by asterisk.

### Effectiveness of joint learning

In Table 4, we compare the performance of the **corpus only** baseline, which does not use a KB for learning word embeddings (corresponds to λ = 0 in Eq 5), against the level of performance we would obtain if we had used both a corpus and a KB. In particular, we study the effect of using 8 different WordNet semantic relations as the default relation type for the KB. We use the XL corpus and learn *d* = 300 dimensional word embeddings using the SKB method for each relation type.

**Table 4:** Effectiveness of joint learning.

Method	RG	MC	RW	SCWS	MEN	SimLex	sem	syn	total	SemEval
corpus only	0.7545	0.6796	0.2522	0.4829	0.7015	0.3274	58.94	65.46	62.50	38.44
Synonyms	**0.7879**	**0.7614**	0.2674	**0.5103**	0.7367*	0.3492	59.90	71.02*	65.97*	**39.39**
Antonyms	0.7687	0.7018	0.2545	0.4907	0.7142	0.3268	59.54	67.07*	63.65*	39.01
Hypernyms	0.7774	0.7330	0.2536	0.5034	0.7335*	0.3576*	60.15*	71.91*	66.57*	39.22
Hyponyms	0.7720	0.7193	0.2616	0.5040	0.7292*	0.3575	60.05*	70.75*	65.89*	39.03
Member-holonyms	0.7655	0.6985	0.2536	0.4869	0.7059	0.3310	59.53	65.91	63.01	38.49
Member-meronyms	0.7613	0.6952	0.2537	0.4867	0.7070	0.3332	58.94	65.68	62.62	38.61
Part-holonyms	0.7740	0.7144	0.2682	0.4937	0.7220*	0.3298	59.10	67.86*	63.89*	38.96
Part-meronyms	0.7814	0.7338	**0.2714**	0.4980	0.7215*	0.3317	59.36	67.65*	63.89*	38.95

From Table 4, we see that by jointly learning with a KB, we can always outperform the **corpus only** baseline, irrespective of the relation type. This result supports our proposal to use both corpora and KBs jointly for learning word embeddings. Among the relation types, synonymy reports the best performance in **RG**, **MC**, **SCWS**, **MEN** and **SemEval** benchmarks, whereas hypernymy reports the best performance in **SimLex** and effective for answering **sem** and **syn** analogy questions in Google dataset. The fact that word similarity benchmarks contain many word pairs that are similar, explains the effectiveness of synonymy. Moreover, part-meronyms, part-meronyms and syonyms are performing well in predicting the semantic similarity between rare words (**RW**), is important because it shows that by incorporating a semantic lexicon we can learn a better embeddings for words that rarely co-occur even in a large corpora [41].

### Effectiveness of dynamic expansion

To compare the word embeddings learnt by the proposed method using the two dynamic KB expansion methods NNE and HNE over SKB, we train word embeddings using each method separately. In Table 5, we compare the results that we obtained by expanding the KB in synonymy relation, which is also the best individual relation type according to the analysis in Section, against the SKB. From Table 5, we can see that both NNE and HNE outperforms SKB in most of the benchmarks. In particular, NNE reports the best performance in **RW**, **SCWS**, **SimLex**, **syn** and **SemEval**, whereas the best scores in **MC** and **MEN** achieved by HNE. However, the differences among the three methods are not statistically significant after one expansion round. As we later discuss in Section, NNE and HNE significantly outperform SKB in various benchmarks when we repeat the expansion process multiple rounds.

**Table 5:** Comparisons among SKB, NNE and HNE using synonym relation type on XL corpus.

Method	RG	MC	RW	SCWS	MEN	SimLex	sem	syn	total	SemEval
SKB	**0.7879**	0.7614	0.2674	0.5103	0.7367	0.3492	**59.9**	71.02	65.97	39.39
NNE	0.7875	0.753	**0.2684**	**0.5128**	0.7390	**0.3535**	59.75	**71.25**	**66.04**	**39.48**
HNE	0.7852	**0.7738**	0.2682	0.5122	**0.7409**	0.3515	59.75	71.02	65.91	39.10

In Table 6, we compare the proposed method against previously proposed word embedding learning methods that use both a corpus and a KB. Specifically, we compare against Relation Constraint Model (**RCM**) [21]. Relational information (**R-NET**), Categorical Information (**C-NET**) and the union of Relational and Categorical (**RC-NET**) [22], and Retrofitting (**Retro**) [10]. Details of those methods are provided in Section.

**Table 6:** Comparisons against prior work.

Method	RG	MC	RW	SCWS	MEN	SimLex	sem	syn	SemEval
RCM	0.471	-	-	-	0.501	-	-	29.90	-
R-NET	-	-	-	-	-	-	32.64	43.46	-
C-NET	-	-	-	-	-	-	37.07	40.06	-
RC-NET	-	-		-	-	-	34.36	44.42	-
Retro (CBOW)	0.577	0.5693	0.2512	0.4764	0.605	0.2718	36.65	52.50	38.22
Retro (SG)	0.745	0.7446	0.2498	0.4813	0.657	0.3911	45.29	65.65	38.74
Retro (corpus only)	0.7865	0.7544	0.2552	0.4802	0.673	**0.3936**	**61.11**	68.14	38.70
SKB (synonyms)	**0.7879**	0.7614	0.2674	0.5103	0.7367*	0.3492	59.90	71.02*	39.39
NNE (synonyms)	0.7875	0.753	**0.2684**	**0.5128**	0.739*	0.3535	59.75	71.25*	**39.48**
HNE (synonyms)	0.7852	**0.7738**	0.2682	0.5122	0.741*	0.3515	59.75	71.02*	39.1

We use the publicly available source codes of **Retro** to retrofit the vectors learnt by CBOW (**Retro (CBOW)**), and skip-gram (**Retro (SG)**). We also retrofit the vectors learnt by the corpus only baseline (**Retro (corpus only)**). All of the above-mentioned methods are trained using ukWaC as the corpus and synonyms extracted from the WordNet as the KB. Unfortunately, the implementations nor trained word embeddings were available for **RCM**, **R-NET**, **C-NET** and **RC-NET** methods. Therefore, for those methods we compare the results reported in the original publications. Consequently, it is noteworthy that **RCM**, **R-NET**, **C-NET** and **RC-NET** are trained with different corpus and KB which can indeed affect the performance. A dash in Table 6 indicates that the performance on that dataset was not reported in the original publication.

From Table 6, we see that the proposed method under NNE obtains the best performance on **RW**, **SCWS**, **syn** and **SemEval**, whereas HNE reports the best performance on **MC** and **MEN**. The **SKB** obtains the best performance on the **RG** dataset, whereas **Retro (corpus only)** reports the best results on the **sem** and **SimLex** datasets.

### Effect of the corpus size

To study effect of the size of the corpus on the performance of the proposed method, we use the five sub-corpora defined in Table 1, and train word embeddings with the complete WordNet KB. We evaluate the trained word embeddings using the benchmark dataset and report results in Table 7. Overall, as prior work has shown [11] [46], Table 7 shows that a larger corpus size helps for obtaining a better level of performance. All the results reported in Table 7 use the synonym relation.

**Table 7:** Performance of the proposed method using SKB, NNE and HNE against the baseline in various corpus sizes with synonym relation.

Method	Corpus Size	RG	MC	RW	SCWS	MEN	SimLex	sem	syn	total	SemEval
corpus only	XL	0.7545	0.6796	0.2522	0.4829	0.7015	0.3274	58.94	65.46	62.50	38.44
SKB		**0.7879**	0.7614	0.2674	0.5103	0.7367*	0.3492	**59.90**	71.02*	65.97*	39.39
NNE		0.7875	0.753	**0.2684**	**0.5128**	0.739*	**0.3535**	59.75	71.25*	66.04*	**39.48**
HNE		0.7852	**0.7738**	0.2682	0.5122	0.7409*	0.3515	59.75	71.02*	65.91*	39.10
corpus only	L	0.7385	0.6238	0.2275	0.4719	0.6950	0.3235	57.37	64.65	61.35	38.19
SKB		0.7698	**0.7231**	0.2363	0.4986	0.7278*	0.3461	58.64*	68.94*	64.27*	38.64
NNE		0.7649	0.7171	**0.2384**	**0.5037**	0.7302*	**0.3504**	58.28	69.10*	64.19*	**38.74**
HNE		**0.7712**	0.7207	0.2375	0.5017	0.732*	0.3481	58.68*	69.07*	64.36*	38.54
corpus only	M	0.7016	0.5899	0.2029	0.4687	0.6892	0.3157	51.71	62.79	57.76	37.5
SKB		**0.7257**	**0.6635**	0.2078	0.4950	0.7187*	0.3327	52.41	65.48*	59.55*	38.18
NNE		0.7217	0.646	**0.2116**	0.4966	0.7211*	**0.3366**	**52.54**	65.55*	59.65*	38.17
HNE		0.7225	0.652	0.210	**0.4981**	0.7226*	0.3342	52.36	65.59*	59.58*	**38.26**
corpus only	S	0.6948	0.5904	0.1681	0.4509	0.6704	0.2978	43.08	56.77	50.56	37.24
SKB		**0.7145**	0.6616	0.1774	0.4740	0.6923*	0.3113	43.27	58.22*	51.44*	37.48
NNE		0.71	0.6287	**0.1782**	**0.4765**	0.6943*	**0.3163**	43.38	58.44*	51.61*	**37.73**
HNE		0.7118	**0.6648**	0.1775	0.4762	0.6963*	0.3126	**43.42**	58.25*	51.52*	37.52
corpus only	XS	0.6408	0.6227	0.1632	0.4446	0.6404	0.2636	31.72	48.99	41.15	36.33
SKB		0.6522	0.6725	0.1759	0.459	0.6565	0.2741	32.01	49.61	41.62	36.58
NNE		0.6529	0.6632	**0.1764**	0.4622	0.6580	**0.2772**	**32.16**	**49.73**	**41.76**	**36.78**
HNE		**0.657**	**0.6726**	0.1739	**0.4624**	**0.6595**	0.2749	32.10	49.69	41.71	36.52

**Fig 1:**
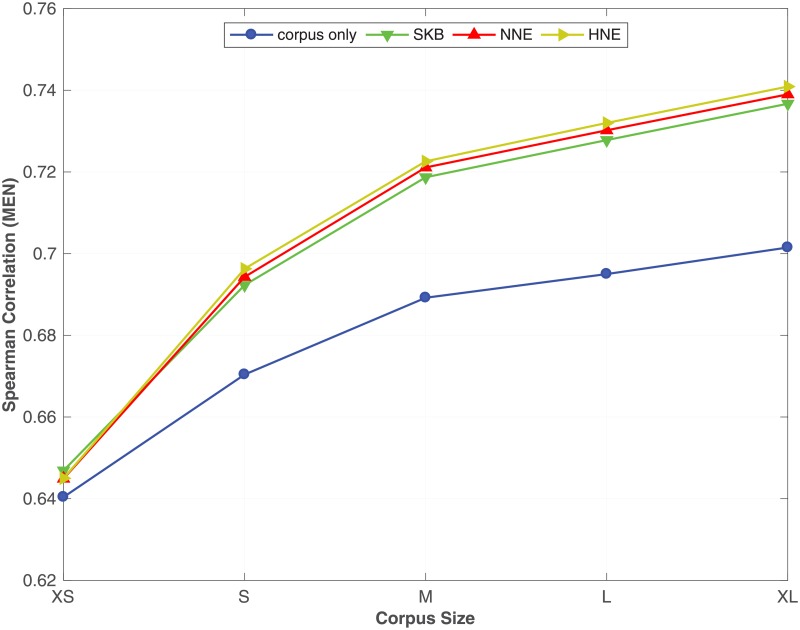
Effect of the corpus size. The effect of varying the size of the corpus under SKB, NNE, HNE on the **MEN** dataset. The full WordNet is used as the KB.

**Fig 2:**
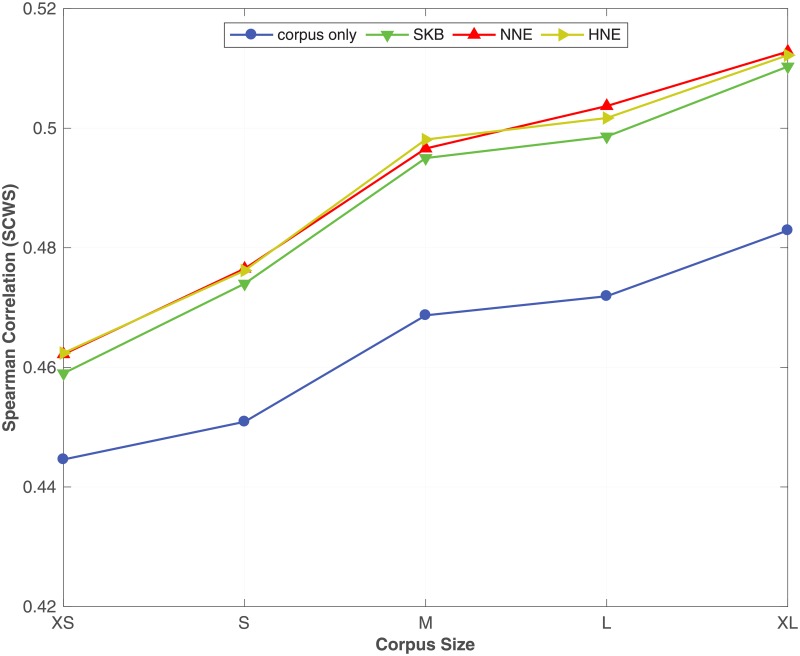
Effect of the corpus size. The effect of varying the size of the corpus under SKB, NNE, HNE on the **SCWS** dataset. The full WordNet is used as the KB.

**Fig 3:**
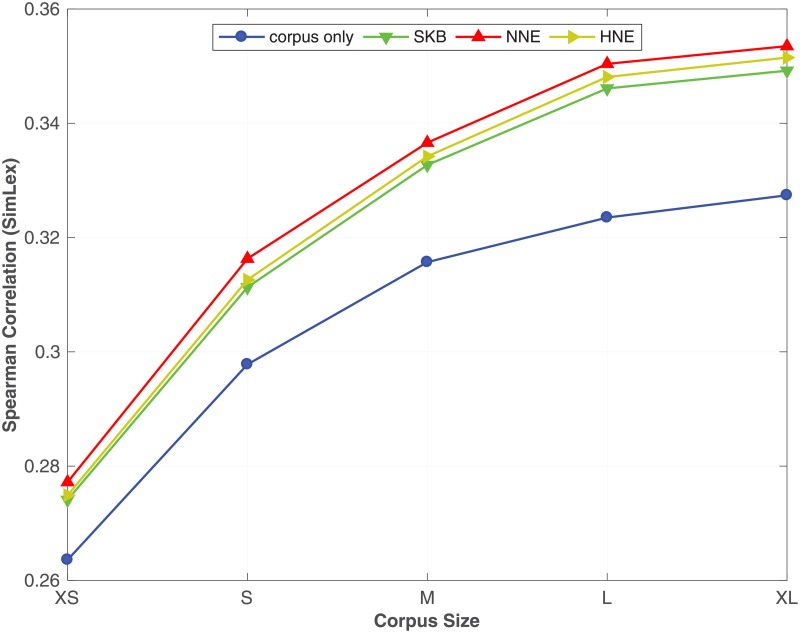
Effect of the corpus size. The effect of varying the size of the corpus under SKB, NNE, HNE on the **SimLex** dataset. The full WordNet is used as the KB.

From Table 7, we see that by incorporating the synonym semantic relation using SKB, NNE and HNE with different corpus sizes, the proposed method always outperforms the corpus only baseline on all benchmark datasets. Moreover, we see that NNE and HNE produce better word embeddings over SKB in most of the benchmark datasets. In particular, NNE and HNE obtain a significant improvement over SKB for predicting similarity between words in **RW**, **SCWS** and **MEN** benchmarks across all different corpus sizes. Moreover, in the word analogy prediction task, NNE and HNE constantly outperform SKB on **syn** and **SemEval** datasets, irrespective of the size of the corpus. To readily understand the effect of the corpus size on the accuracy of the word embeddings learnt by the proposed method, in Figs 1, 2 and 3, we plot the Spearman correlation coefficient against the size of the corpus for respectively **MEN**, **SCWS** and **SimLex** datasets. We selected **MEN**, **SCWS** and **SimLex** here because those datasets have the largest numbers of word-pairs among all the word similarity benchmark datasets. We can clearly see that irrespective of the size of the corpus, it is always beneficial to combine the corpus with the KB to learn higher-quality word embeddings, whereas the differences between the different expansion methods are relatively small.

### Effect of the KB size

**Fig 4:**
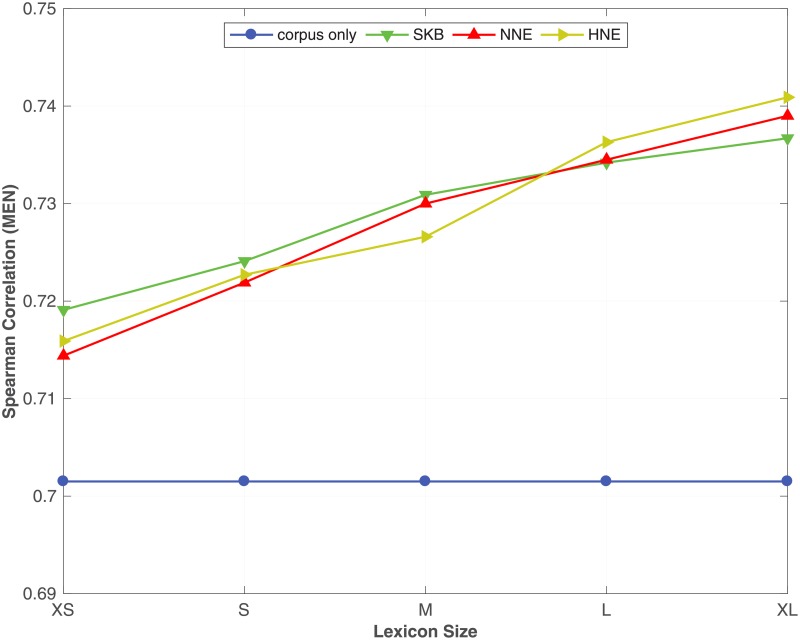
Effect of the KB size. The effect of using different lexicon (synonym relation) sizes on the proposed method with SKB, NNE and HNE evaluated on **MEN** dataset. The full ukWaC corpus is used as the corpus.

**Fig 5:**
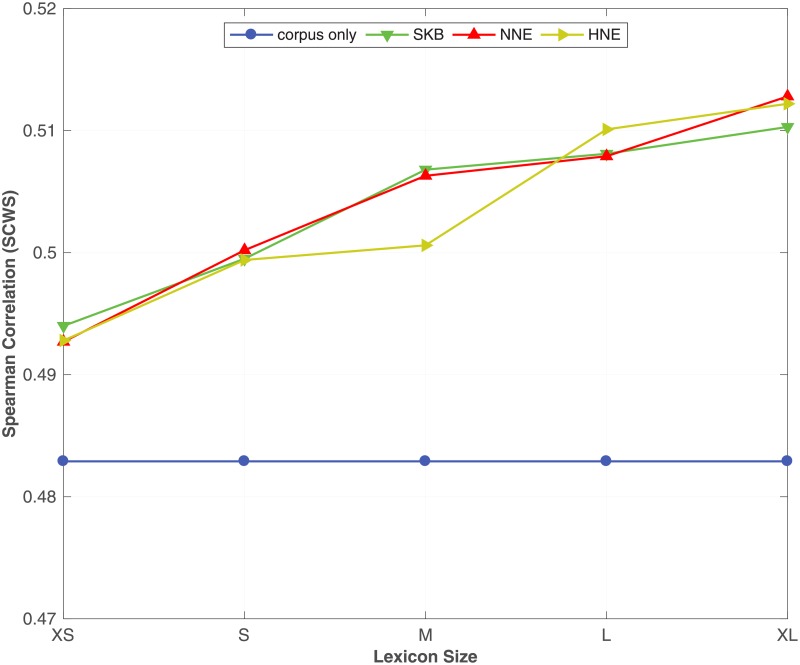
Effect of the KB size. The effect of using different lexicon (synonym relation) sizes on the proposed method with SKB, NNE and HNE evaluated on **SCWS** dataset. The full ukWaC corpus is used as the corpus.

**Fig 6:**
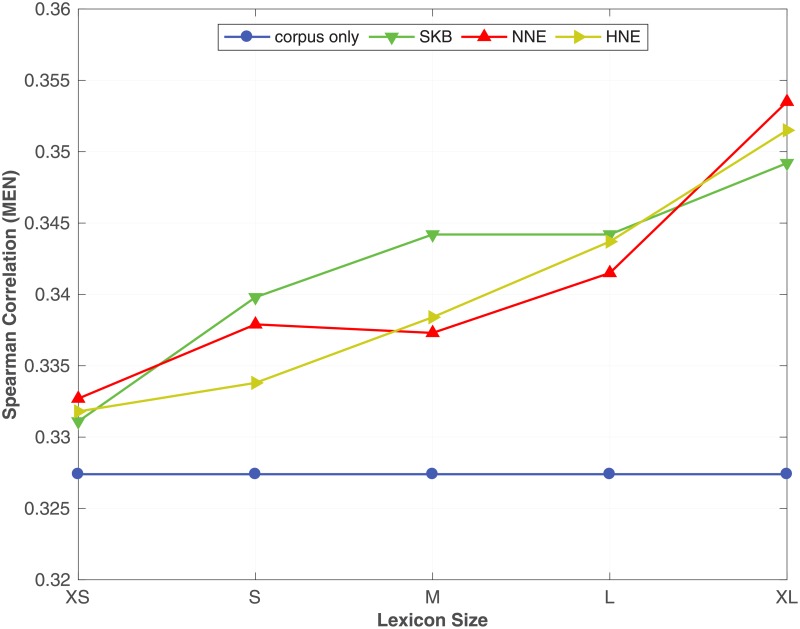
Effect of the KB size. The effect of using different lexicon (synonym relation) sizes on the proposed method with SKB, NNE and HNE evaluated on **SimLex** dataset. The full ukWaC corpus is used as the corpus.

To evaluate the impact of the size of the KB on the proposed method, we randomly select pairs of synonyms from WordNet synsets to create KBs of varying sizes as shown in Table 8. We jointly train with each KB and the entire ukWaC corpus. Figs 4, 5 and 6 show the impact of varying the semantic lexicon size on the proposed method evaluated respectively on **MEN**, **SCWS** and **SimLex** benchmarks. Similar trends were also observed with other benchmark datasets. We fixed the corpus size with XL and perform the experiments with various lexicon sizes. The horizontal lines in the three figures (Figs 4, 5 and 6) correspond to the **corpus-only** baseline, which is unaffected when the corpus is not varied. We see that the proposed method using **SKB**, **NNE**, and **HNE** continuously increase performance when we increase the size of the KB. This result suggests that we can still learn high-quality word embeddings by creating KBs with better coverage on top of what we can learn about word semantics from a large corpora. **HNE**, unlike **NNE**, requires expansion candidates to be mutual neighbours. With smaller KB, it is difficult to find such mutual neighbours, which results in **HNE** performing poorly compared to **SKB** and **NNE**. However, when we increase the size of the KB, **HNE**’s performance increases.

**Table 8:** Different semantic lexicon sizes (synonym relation) randomly selected from WordNet.

% of synonym word-pairs	Edges			Size
	SKB	NNE	HNE	
100	87,060	108,510	104,123	XL
70	60,941	75,957	72,886	L
40	34,824	43,404	41,649	M
20	17,412	21,702	20,824	S
10	8,706	10,851	10,412	XS

### Multi-rounds of expansion

The **NNE** (Algorithm 2) and **HNE** (Algorithm 3) methods can be repeatedly used to expand a KB using the word embeddings learnt from previous rounds. Specifically, once we have expanded the KB using either **NNE** or **HNE**, we run Algorithm 1 with the same settings T = 20 and λ = 10,000 to learn word embeddings. Next, we use those word embeddings to find the nearest neighbours used in **NNE** and **HNE**. We then expand the KB using Algorithms 2 or 3. Because the word embeddings learnt after expanding the KB could be better than the original word embeddings, by using the newer word embeddings we can hope to find more nearest neighbours, thereby further expanding the KB. Similar to all the above experiments in the previous sections, we use the WS dataset as validation data for tuning the number of expanding rounds. We observed that 10 rounds were sufficient where with further expansion the performance start falling behind the SKB baseline. We also observed that 5 rounds represent, on average, the peak point for most of the benchmark datasets. In Table 9, we compare the results that we obtained by expanding the KB with 5 rounds of expansion in all the 8 different WordNet semantic relations against the SKB. From Table 9, we can see that both NNE and HNE outperforms SKB in most of the benchmarks irrespective of the relation types. In particular, NNE on synonyms, hypernyms, part-holonyms and part-meronyms reports the best performance on most of the benchmarks, whereas HNE works better on hyponyms, member-holonyms and member-meronyms.

**Table 9:** Comparisons among SKB, NNE and HNE using different relation types with 5 expansion rounds on XL corpus.

Method	Relation	RG	MC	RW	SCWS	MEN	SimLex	sem	syn	total	SemEval
SKB	synonyms	0.7879	0.7614	0.2674	0.5103	0.7367	0.3492	59.9	71.02	65.97	39.39
NNE		**0.7896**	0.7552	**0.2706**	**0.5281**	0.7434	**0.3651**	59.98	**71.44**	**66.24**	**39.52**
HNE		0.7883	**0.7745**	0.2694	0.5198	**0.7436**	0.3627	**60.1**	71.36	66.21	39.23
SKB	antonyms	**0.7687**	0.7018	0.2545	0.4907	0.7142	0.3268	59.54	67.07	63.65	39.01
NNE		0.7668	0.7022	0.2553	0.5106	0.7166	**0.3284**	**59.86**	67.24	63.89	**39.07**
HNE		0.7682	**0.7029**	**0.2561**	**0.5114**	**0.7169**	0.3275	59.79	**67.36**	**63.92**	39.05
SKB	hypernyms	0.7774	0.7330	0.2536	0.5034	0.7335	0.3576	**60.15**	71.91	66.57	39.22
NNE		**0.7792**	**0.7392**	0.2543	**0.5162**	0.7372	**0.3647**	60.13	**72.71**	**67.02**	**39.36**
HNE		0.7724	0.7043	**0.2554**	0.5122	**0.7385**	0.3633	60.14	72.63	66.96	39.28
SKB	hyponyms	0.7720	0.7193	0.2616	0.5040	0.7292	0.3575	60.05	70.75	65.89	38.49
NNE		0.7738	**0.7214**	0.2633	0.5105	0.7318	0.3582	**60.22**	**70.83**	**66.02**	**39.31**
HNE		**0.7771**	0.7193	**0.2645**	**0.5109**	**0.7336**	**0.3583**	62.2	70.79	65.99	39.22
SKB	member holonyms	0.7655	0.6985	0.2536	0.4869	0.7059	0.3310	59.53	65.91	63.01	38.49
NNE		**0.7698**	**0.7067**	0.2546	0.4882	0.7072	**0.3368**	59.64	66.09	63.17	38.64
HNE		0.7671	0.7015	**0.2551**	**0.4897**	**0.7096**	0.3339	**59.71**	**66.16**	**63.23**	**38.72**
SKB	member meronyms	0.7613	0.6952	0.2537	0.4867	0.7070	0.3332	58.94	65.68	62.62	38.61
NNE		**0.7644**	**0.6988**	**0.2555**	**0.4895**	0.7092	**0.3355**	59.28	65.92	62.92	**38.88**
HNE		0.7637	0.6973	0.2547	0.4891	**0.7093**	0.3354	**59.38**	**65.97**	**62.99**	38.72
SKB	part holonyms	0.7740	0.7144	0.2682	0.4937	0.7220	0.3298	59.10	67.86	63.89	38.96
NNE		**0.7791**	**0.7264**	0.2688	**0.5019**	0.7266	**0.3325**	59.24	67.92	63.98	**39.27**
HNE		0.7782	0.7252	**0.2694**	0.5002	**0.7269**	0.3316	**59.31**	**67.95**	**64.03**	39.18
SKB	part meronyms	0.7814	0.7338	0.2714	0.4980	0.7215	0.3317	59.36	67.65	63.89	38.95
NNE		**0.7854**	0.7349	**0.2758**	**0.5028**	0.7237	0.3328	**59.64**	**67.97**	**64.19**	**39.26**
HNE		0.7822	**0.7352**	0.2742	0.5016	**0.7252**	**0.3334**	59.45	67.75	63.98	39.18

**Fig 7:**
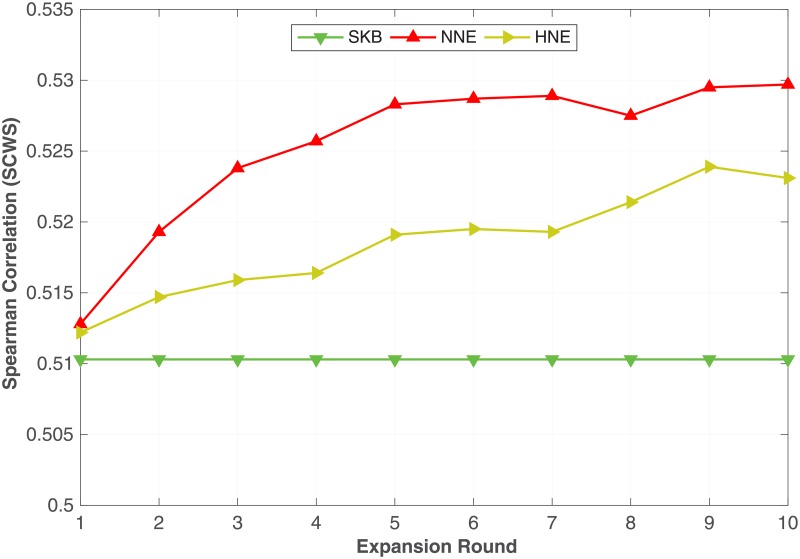
Multi-rounds of expansion. The impact of multi-rounds of expansion using NNE and HNE with synonym relation evaluated on the **SCWS** dataset.

**Fig 8:**
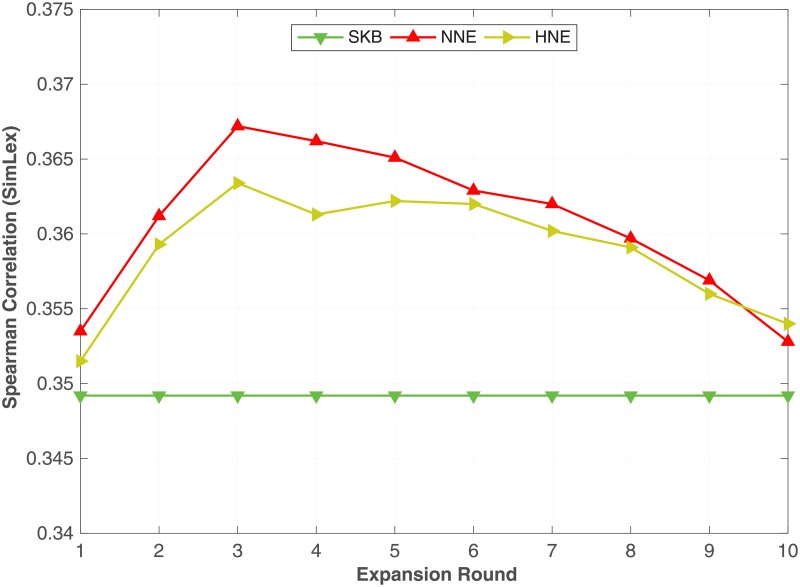
Multi-rounds of expansion. The impact of multi-rounds of expansion using NNE and HNE with synonym relation evaluated on the **SimLex** dataset.

To readily understand the impact of the multi-rounds of expansion, in Figs 7 and 8, we plot the Spearman correlation coefficient on **SCWS** and **SimLex** datasets against the number of expansion rounds with **NNE** and **HNE**.

The horizontal line corresponds to the **SKB** method that does not expand the KB. From the same figures, we can see that on Fig 7 (**SCWS**) for both **NNE** and **HNE**, the performance increases with the number of expansion rounds, until approximately the 9-th round, where the performance saturates. Whereas on Fig 8 (**SimLex**) the performance reaches its peak earlier on the 3-rd round for both **NNE** and **HNE** and steadily decreases until approximately the 10-th round where the performance starts falling behind the SKB baseline. Similar trends were observed in all benchmark datasets, where multi-round expansion improves performance over single-round expansion in all cases but the performance either saturates or degrades because more noisy and irrelevant expansion candidates are introduced in later expansion rounds. Similar trends have been observed in bootstrapping methods for relation or entity extraction [47]. Determining the ideal number of rounds and preventing noisy expansions require further research.

### Effect of dimensionality

**Fig 9:**
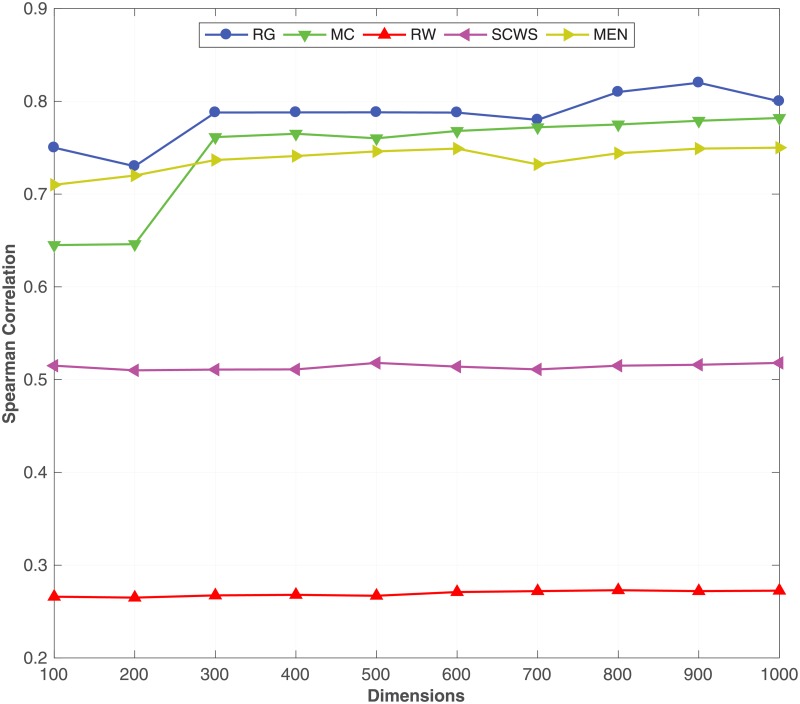
Effect of dimensionality. The impact of the dimensionality of the word embeddings learnt evaluated on the **RG**, **MC**, **RW**, **SCWS** and **MEN** datasets.

To study the impact of the dimensionality *d* on the performance of the proposed method, we train word embeddings with different dimensionalities using ukWaC as the corpus and synonymy relation on WorNet as the KB. Fig 9 shows the performance on the semantic similarity benchmark datasets. We can see from the same figure that even with a wide range of dimensionalities the proposed method reports a relatively stable performance. Interestingly, with as small as 300 dimensions, we can capture semantics of words, corresponding to prior work [24] [11]. Importantly, Fig 9 shows that adding more dimensions does not result in any decrease in the performance due to overfitting, which is encouraging.

## Conclusion

We proposed a method that utilises the information contained in KBs to learn a better word embeddings as compared to corpus-only approaches. In particular, we use the corpus to define a learning objective subject to the constraints extracted from the KB. Moreover, we proposed two methods for expanding the KB using information extracted from the corpus, for the purpose of learning high-quality word embeddings. Our experimental results on a range of benchmark datasets for semantic similarity and word analogy show that the proposed method obtains improvements over a **corpus-only** word embedding learning methods, and previously proposed joint word embedding learning methods. Furthermore, empirical experiments conducted with varying sizes of corpora and KBs show that the proposed method reports consistent improvements over a wide range of different configurations of resources. Interestingly, by repeatedly expanding the KB, we can further improve the accuracy of the learnt word embeddings. In future, we plan to apply the proposed method to learn word embeddings from different types of KBs such as medical or legal ontologies.

## Appendix

The gradients of the objective given by Eq 5 w.r.t. the four variables are computed as follows:

$$\begin{array}{rr} \frac{\partial J}{\partial w_{i}}= & \sum_{j}f\left(X_{ij}\right){\tilde{w}}_{j}\left(w_{i}{}^{⊤}{\tilde{w}}_{j}+b_{i}+{\tilde{b}}_{j}-\text{log}\left(X_{ij}\right)\right)\\ & +\lambda \sum_{j}R\left(w_{i},w_{j}\right)\left(w_{i}-{\tilde{w}}_{j}\right) \end{array}$$ (8)

$$\begin{array}{rr} \frac{\partial J}{\partial b_{i}}= & \sum_{j}f\left(X_{ij}\right)\left(w_{i}{}^{⊤}{\tilde{w}}_{j}+b_{i}+{\tilde{b}}_{j}-\text{log}\left(X_{ij}\right)\right) \end{array}$$ (9)

$$\begin{array}{rr} \frac{\partial J}{\partial {\tilde{w}}_{j}}= & \sum_{i}f\left(X_{ij}\right)w_{i}\left(w_{i}{}^{⊤}{\tilde{w}}_{j}+b_{i}+{\tilde{b}}_{j}-\text{log}\left(X_{ij}\right)\right)\\ & -\lambda \sum_{j}R\left(w_{i},w_{j}\right)\left(w_{i}-{\tilde{w}}_{j}\right) \end{array}$$ (10)

$$\begin{array}{rr} \frac{\partial J}{\partial {\tilde{b}}_{j}}= & \sum_{i}f\left(X_{ij}\right)\left(w_{i}{}^{⊤}{\tilde{w}}_{j}+b_{i}+{\tilde{b}}_{j}-\text{log}\left(X_{ij}\right)\right) \end{array}$$ (11)
